# Supplementary material for: Chromothripsis during telomere crisis is independent of NHEJ, and consistent with a replicative origin
Source: Genome Res. 2019 May;29(5):737–49. doi: 10.1101/gr.240705.118 (PMC6499312; doi:10.1101/gr.240705.118)
Supplement: Supplemental Material [file supp_gr.240705.118_Supplemental_file_1.zip › contigs/annotated_contigs/DB51/contig.2.DB51_length_624_mean_cov_3.92467948718.docx]

**DB51_length_624_mean_cov_3.92467948718**

```
GTGTATGTGTATGTGTGTGTGTCACTTAACAATTAATCTTCAAATTCCTAAAGAAAATAAAAGGACTAAAAATATCACCTCTCCTCCCT
 >chr6:80951914-80952281 - E=4e-209
GCTCCCACCCCACCAAAAATAACTGAAGAAGAGCAAGTTCGGGAGATTTGCCCTATCAGATATTCAAATTTATTATAAAGCTATATTGT

TAAGATAGAGAGGTATTGTAAGGCTCTAAAAAGACACAAAGAAACATAAAATGGACAGCCCATGAACAAATCCACGTGTACATGAACAG

TGGATACTGTTAAAAGATGCAGAGTGTAGGGTCAAAAATAAAACAGAGCTCTTACCTTAAATCACCCTAAATATATCAAGTATGATTAA

AGGTGACT|AGA|AATGGGTGTTGCTTGGCCAATCTTAGTAACTAATGGATGTGGTCGGAAAAACAGAGAACGTGGAGATTCCCAGAAG
         >chr6:158066867-158067125 - E=2e-140
AGAAACGTGCAGAACGGCAGGTGGAGAAGTTTCCACGGCACCGTCTCTGTTCCCAAGGGCTCACATCTGGAACGGGATGTATGGGAAAA

GCTGGGCTCAACCTTTTCTTTCCTTTTTCTCCTCCATTGCCCCAAACCCAAGAACACGCCCGTGTCCCGTGTGGTCAGGAACTTACGTC

C|CC
```
